# Disruption of IL-17-mediated immunosurveillance in the respiratory mucosa results in invasive *Streptococcus pyogenes* infection

**DOI:** 10.3389/fimmu.2024.1351777

**Published:** 2024-03-21

**Authors:** Jamie-Lee Mills, Ailin Lepletier, Victoria Ozberk, Jessica Dooley, Jacqualine Kaden, Ainslie Calcutt, Yongbao Huo, Allan Hicks, Ali Zaid, Michael F. Good, Manisha Pandey

**Affiliations:** ^1^ Institute for Glycomics, Griffith University, Gold Coast, QLD, Australia; ^2^ School of Pharmacy and Medical Sciences, Griffith University, Gold Coast, QLD, Australia

**Keywords:** *S. pyogenes*, IL-17, invasive streptococcal infection, mucosal infection, natural immunity

## Abstract

**Introduction:**

*Streptococcus pyogenes* is a Gram-positive pathogen that causes a significant global burden of skin pyoderma and pharyngitis. In some cases, infection can lead to severe invasive streptococcal diseases. Previous studies have shown that IL-17 deficiency in mice (IL-17^−/−^) can reduce *S. pyogenes* clearance from the mucosal surfaces. However, the effect of IL-17 on the development of severe invasive streptococcal disease has not yet been assessed.

**Methods:**

Here, we modeled single or repeated non-lethal intranasal (IN) *S. pyogenes* M1 strain infections in immunocompetent and IL-17^−/−^ mice to assess bacterial colonization following a final IN or skin challenge.

**Results:**

Immunocompetent mice that received a single *S. pyogenes* infection showed long-lasting immunity to subsequent IN infection, and no bacteria were detected in the lymph nodes or spleens. However, in the absence of IL-17, a single IN infection resulted in dissemination of *S. pyogenes* to the lymphoid organs, which was accentuated by repeated IN infections. In contrast to what was observed in the respiratory mucosa, skin immunity did not correlate with the systemic levels of IL-17. Instead, it was found to be associated with the activation of germinal center responses and accumulation of neutrophils in the spleen.

**Discussion:**

Our results demonstrated that IL-17 plays a critical role in preventing invasive disease following *S. pyogenes* infection of the respiratory tract.

## Introduction

*Streptococcus pyogenes* (Group A Streptococcus) colonizes the upper respiratory tract (URT) and skin. In some cases, the mucosal and skin barriers become vulnerable to bacterial escape, leading to invasive infections. These infections include potentially life-threatening conditions, such as sepsis, pneumonia, necrotizing fasciitis, and toxic shock syndrome. *S. pyogenes* infection can also lead to post-streptococcal autoimmune diseases, primarily acute rheumatic fever and rheumatic heart disease (RHD). Collectively, streptococcus-related pathologies are responsible for the loss of approximately 500,000 lives each year (1, 2), with the greatest burden experienced by people in developing countries and indigenous populations living in economically advanced societies (3). The development of natural immunity to *S. pyogenes* at the primary site of infection is slow, and a vaccine is not yet available.

Antibodies (IgA and IgG) (4–6), effector immune cells (CD4^+^ T cells, macrophages, and neutrophils) (7–9) and cytokines (including IL-17A and IFN-ɣ) (7, 8, 10) have been shown to play critical roles in regulating immune responses to *S. pyogenes* at the site of infection. These immune responses collectively target multiple streptococcal antigens, with a key antigen being the major virulence factor, the M-protein (encoded by the *emm* gene). In addition to the M protein, other bacterial virulence factors have also been shown to suppress innate and acquired immune responses in *S. pyogenes* infection (11). However, the immune mechanisms that facilitate systemic dissemination of *S. pyogenes* from the respiratory mucosa and skin remain elusive.

Early streptococcal research in humans demonstrated that protection against homologous strains following pharyngeal infection is long lasting. M-type-specific antibodies were recovered in convalescent blood following pharyngitis (12, 13), with bacteriostatic properties persisting in some individuals (13) and remaining present in the blood for a substantial length of time. The longest duration reported by Lancefield was up to 32 years (14). While earlier studies focused on infections of the URT, later studies in First-Nation Australian communities, where skin infections are far more prevalent than pharyngitis, identified different strains moving through communities. In some cases, more than one strain existed at a time and persisted for longer than 6 months (15). Thus, it is likely that *S. pyogenes* strains will remain in the community as a reservoir of skin infection. In tropical communities with high rates of RHD, immunity to a skin strain of *S. pyogenes* is slow to develop (15). In agreement with these clinical findings, our previous study using a murine model of invasive streptococcal disease associated with skin pyoderma showed that immunity in the skin and spleen required repeated homologous skin infections (5). In that study, enduring protection was correlated with M-type-specific memory B-cell responses in the sera, spleen, and bone marrow, in the absence of which immunity was rapidly lost (5).

While systemic immunity to *S. pyogenes* infection is associated with IgG responses in the sera, mucosal immunity relies on the production of secretory IgA (which can prevent the attachment of *S. pyogenes* to mucosal surfaces (16)), and IL-17 (which results in recruitment of neutrophils and other inflammatory cells that contribute to bacterial clearance at mucosal sites (17, 18). Inborn IL-17 deficiency and therapeutics based on IL-17 inhibitors increase the risk of mucocutaneous candidiasis in humans (19–21). Similarly, the most common adverse effect of anti-IL-17 therapy in patients with psoriasis is an increase in nasopharyngeal tract infections (22).

Besides *S. pyogenes*, the route of infection by other bacteria can cause fundamental differences in the resulting immune responses (23). Intranasal *Francisella tularensis* infection induces a Th17 response in the lungs, whereas the intradermal route of infection favors a Th1 response in both the spleen and lungs (24). Therefore, immunity at the mucosal, skin, and systemic sites may be regulated separately. These diverse immune responses may impede the development of resistance to infection in cases where an organism can infect via different anatomical sites.

In this study, we investigated the development of mucosal and systemic immunity following single or multiple URT infections with a homologous *S. pyogenes* isolate and correlated it with immune mechanisms underpinning site-specific or cross-compartmental protection. Through the assessment of both humoral and cellular immune responses and via the use of IL-17 deficient mice, we showed that CD4^+^ T cells, IL-17, and neutrophil responses in the lungs collectively regulate the protection of the respiratory mucosa. In the absence of IL-17, URT infections resulted in the passage of *S. pyogenes* from mucosal sites into the lymph nodes and spleen. This study provides critical insights into the role of IL-17 in orchestrating the interplay between immune cells in the respiratory mucosa and lymphoid organs, highlighting the importance of integrating strategies that are capable of inducing IL-17 responses alongside humoral responses in the design of vaccines.

## Results

### A single *S. pyogenes* IN infection results in enduring immunity at the respiratory tract

**Figure 1:**
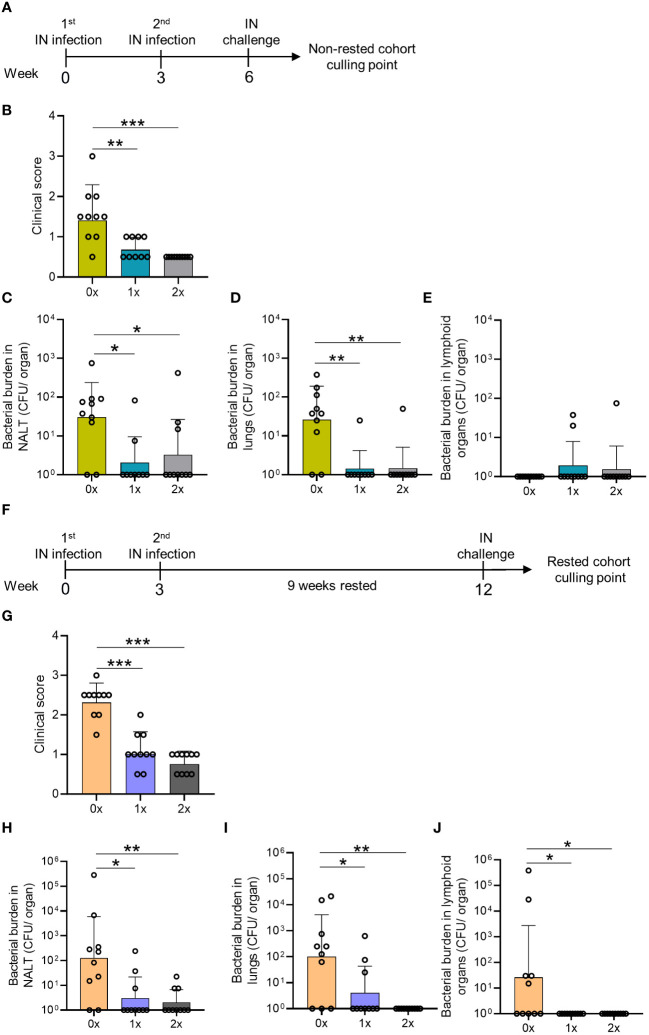
Assessment of mucosal protection and endurance following 0–2 intranasal homologous infections prior to IN challenge. **(A)** BALB/c mice (n = 10, female, 4–6 weeks old) were administered IN infections with 2031 (*emm*1), 3 weeks apart. All mice were subjected to a homologous IN challenge. **(B)** On day 2 following the challenge, the mice were assessed for clinical scores as per the approved score sheet. The bacterial load in **(C)** NALT, **(D)** lungs, and **(E)** lymphoid organs (spleen and pooled cervical lymph nodes). **(F)** BALB/c mice (n = 10, female, 4–6 weeks old) administered IN infections with 2031 were rested for 9 weeks before receiving a homologous IN challenge. **(G)** Blinded clinical scores were assessed on days 1 and 2 following the challenge and are shown as averages. Mice were sacrificed on day 2 post IN challenge to assess the bacterial load in **(H)** NALT, **(I)** lung, and **(J)** lymphoid organs. Data are shown as the CFU geometric mean ± geometric SD. Significance was determined using Mann–Whitney rank analysis of CFU in the sequentially infected group compared to CFU in the challenge control (0×), *p <0.05, **p <0.01, ***p <0.001. The x-axis shows the number of infections before the challenge. 1× only received infection at week 3 and 2× at weeks 0 and 3. The mice were rested for 9 weeks and challenged at week 12. 0× received only the challenge.

We initially sought to demonstrate immunity in the respiratory tract following an intranasal (IN) infection. Cohorts of BALB/c mice received either one or two non-lethal IN infections with mouse-passaged *S. pyogenes* 2031 (*emm*1), each 3 weeks apart (**Figure 1A**). To assess whether the number of prior intranasal exposures would determine the level of site-specific protection, mice received a final IN challenge with homologous *S. pyogenes* isolate three weeks following a single (1x) or two (2x) sequential infections infections. Mice were closely monitored for the appearance of clinical symptoms and scored based on an approved clinical scoring system (25). All mice, regardless of the number of prior IN exposures (1× or 2×), showed a significant reduction (p <0.01-0.001) in clinical scores when compared to the control group, which was not infected prior to challenge (**Figure 1B**). On day 2 post-challenge, mice were euthanized to assess bacterial burden (colony forming units [CFU]) in the nasal-associated lymphoid tissue (NALT) and in the lungs. Following 1x or 2x prior exposures, all mice had significant reductions in bacterial burden in the NALT (66.6%–92.6%; p <0.05) and lungs (90%–100%; p <0.01) when compared to the control group (0×) (**Figures 1C, D**). IN challenge of these mice resulted in a very low to undetectable bacterial burden in lymphoid organs (including spleen and cervical lymph nodes (CLN)), which was comparable across all groups, with or without prior exposure (**Figure 1E**).

To assess the longevity of protection in the respiratory tract, a separate cohort of mice (also previously infected 1x or 2x with *S. pyogenes*) was rested for 9 weeks before receiving a homologous IN challenge (**Figure 1F**). We observed that 1x or 2x infections (**Figures 1A–D**) induced enduring immunity, which did not wane after a 9-week rest period. All mice previously infected with *S. pyogenes* demonstrated a significant reduction in clinical scores compared to control mice (p <0.001) (**Figure 1G**). This was associated with significantly reduced bacterial burden in the NALT (96.3%–99.4%, p <0.05–0.01) and lungs (93.8%–99%, p <0.05–0.01) (**Figures 1H, I**, respectively). Interestingly, IN challenge in mice not previously infected led to bacterial dissemination to lymphoid organs only in the older naive mice (**Figure 1J**), which presented a higher burden in the CLN and spleen, than 9-weeks younger naive mice (**Figure 1E**) (>99.9%, p <0.05), suggesting that age was associated with reduced innate protection. All mice with prior *S. pyogenes* infection demonstrated complete protection against dissemination to the CLN and spleen, which was significantly reduced (>99.9%, p<0.05) compared to the control group (0x) (**Figure 1J**).

Overall, we found that a single IN exposure to *S. pyogenes* resulted in immunity in the respiratory mucosa. Furthermore, our data show age-related susceptibility to bacterial dissemination following *S. pyogenes* IN challenge; however, this was completely prevented by previous exposure to the organism.

### Mucosal immunity against *S. pyogenes* is associated with local humoral and cellular responses

To investigate the role of humoral immunity in mucosal protection, we measured the levels of M-protein type-specific IgG in the sera of mice following each sequential infection. *S. pyogenes* M1 type-specific IgG antibodies were not evident following a single IN infection (**Supplementary Figure 1A**). Nevertheless, M1-specific IgG titers developed after two infections and remained consistent in cohorts that received subsequent IN infections (**Supplementary Figure 1A**).

**Figure 2:**
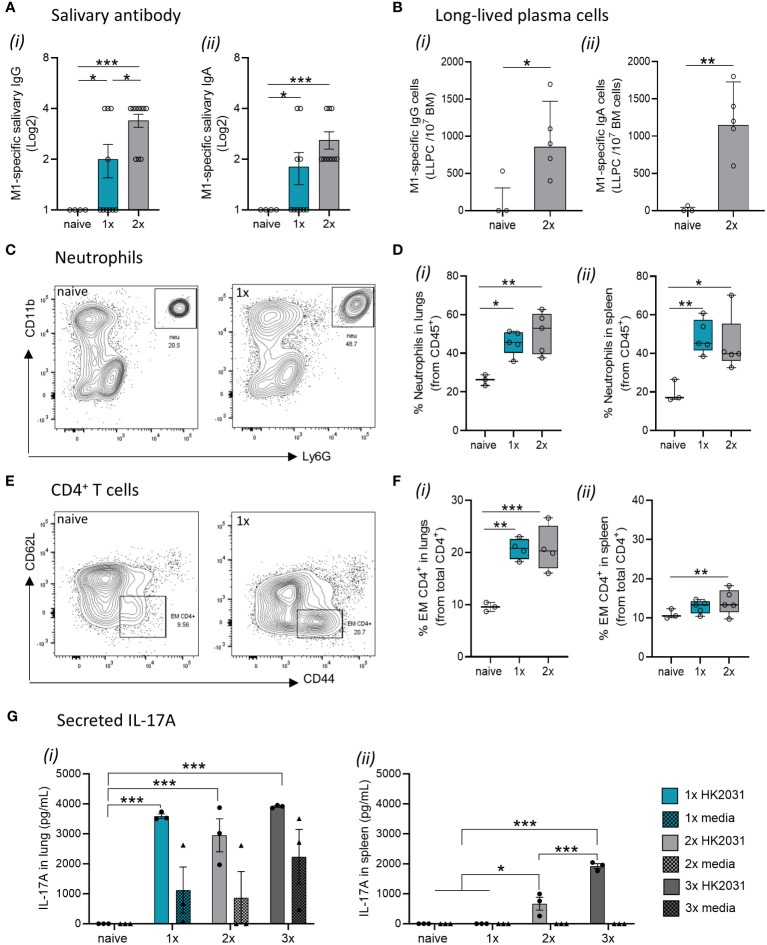
Immune mechanisms involved in mucosal protection. BALB/c mice (n = 5–10 female, 4–6 weeks old) were given a 1x or 2x infections with 2031 (*emm*1), each 3 weeks apart. Three weeks later, all mice received a homologous IN challenge. Mice were sacrificed on day 2 post IN challenge, and organs were homogenized to produce single-cell suspensions for downstream analysis. **(A)** Assessment of salivary **(i)** IgG and **(ii)** IgA using ELISA. End point titers were defined as the highest dilution of saliva for which the OD was >3 standard deviations above the mean OD of control saliva. Significance was determined using One-way ANOVA comparing each group against each other, *p <0.05, ***p <0.001. **(B)** Quantification of long-lived plasma cells in bone-marrow. M1-specific **(i)** IgG and **(ii)** IgA long-lived plasma cell (LLPC) in the bone marrow were enumerated by ELISpot using 3–5 mice/group and are presented as LLPC per 10^7^ bone marrow (BM) cells. Data shown are geometric mean ± geometric SD minus cell counts from naive group [to remove background]). Statistical analysis was performed using Mann–Whitney rank analysis, *p <0.05, **p <0.01. **(C, D)** Neutrophils in lungs and spleen. **(C)** Representative contour plots from flow cytometry analysis of neutrophils (CD45^+^CD11b^+^Ly6G^+^) from the lungs of naïve mice and mice receiving one IN infection with 2031. **(D)** Percentage (%) of neutrophils from CD45^+^ immune cells in the **(i)** lungs and **(ii)** spleen are shown. Data are shown as box-and-whisker. **(E, F)** CD4^+^ T cell in the lungs and spleen. **(E)** Representative contour plots of CD4^+^ T cell memory populations from the lungs of naïve mice and mice receiving one IN infections with 2031. **(F)** Percentage (%) of effector/memory (EM, CD62L^−^CD44^+^) from CD4^+^ T cells (CD3^+^CD4^+^) cells in the **(i)** lungs and **(ii)** spleen are shown. Significance was determined using One-way ANOVA comparing each group against each other *p <0.05, **p <0.01, ***p <0.001. **(G)** IL-17 production by lungs and spleen cells. IL-17A responses were assessed in mice that received a 1x, 2x or 3x infections. Cell isolates from **(i)** lungs and **(ii)** spleens were stimulated *ex vivo* with heat killed 2031 (shown as circle) or media as negative control (shown as triangle). At 72 h post-stimulation, supernatants were isolated, and concentrations of secreted IL-17A was determined using ELISA. Data are presented as pg/mL mean ± SEM. Statistical analysis was performed using Mann–Whitney rank analysis, *p <0.05, **p <0.01, ***p <0.001. X-axis shows the number of infections prior to challenge. 1× only received infection at week 3, 2× at both week 3 and 6 and 3× at weeks 0, 3, and 6. Naïve mice are uninfected controls that did not receive infection or challenge.

The fact that M1-specific circulating antibodies were not evident in mice after a single *S. pyogenes* infection prompted us to investigate other immune correlates of protection in the respiratory mucosa. Salivary antibodies play important roles in protecting against *S. pyogenes* by preventing bacterial attachment to the mucosal epithelia, opsonizing the bacteria, and directly lysing them (26, 27). To investigate URT mucosal immunity, we measured the levels of M1-specific IgG and IgA in the saliva of the mice following each infection. M1-specific IgG and IgA titers were significantly increased in the saliva after a single infection (**Figures 2Ai, ii**, respectively). A progressive increase in M1-specific IgG and IgA responses in saliva of 2x infected mice was also noted. To investigate the long-term humoral immunity mediated by antibody-secreting cells in the bone marrow (BM) (known as long-lived plasma cells (LLPCs)) (28, 29), we counted the number of M1-specific IgG-secreting LLPC in naive and infected mice using an ELISpot assay. We found that the number of M1-specific IgG- and IgA-secreting BM cells was significantly higher in mice that received 2x IN infections than in naïve mice (**Figures 2Bi, ii**, respectively).

Next, to explore the role of effector cell-mediated responses in immunity to *S. pyogenes*, we assessed specific cell populations in the lungs and spleens of sequentially infected mice using flow cytometry. Mice that received either 1x or 2x IN infections had a significant increase in Ly6G^+^ neutrophils in both the lungs (**Figures 2C, Di**) and spleen (**Figure 2Dii**) when compared to naïve mice. Similarly, an increase in effector/memory CD4^+^ T cells was observed in mice that received 1x or 2x infections prior to an IN challenge (**Figures 2E, F**).

To assess whether infection site-specific IL-17 secretion was associated with immunity in the URT, we measured IL-17 secreted by lung cells and splenocytes from mice that received 1x, 2x or 3x IN infections or remained as naive infections. Immune cells isolated from the lungs and spleens were stimulated *ex vivo* with heat-killed (HK) homologous *S. pyogenes* 2031 prior to the detection of IL-17 in the culture supernatant by ELISA. Mice that received 1x, 2x or 3x IN infections produced significantly higher (p <0.001) levels of IL-17 in the lungs than to naïve mice (**Figure 2Gi**); however, at least 2x IN infections were required for induction of IL-17 in the spleen (**Figure 2Gii**).

Taken together, these data show that mucosal immunity following a single *S. pyogenes* IN infection is associated with an increase in M1-specific IgG and IgA antibodies in the saliva, alongside the expansion of neutrophils, effector/memory CD4^+^ T cells, and IL-17^+^ cells in the lung.

### Disruption of IL-17 signaling results in *S. pyogenes* dissemination from the respiratory mucosa

**Figure 3:**
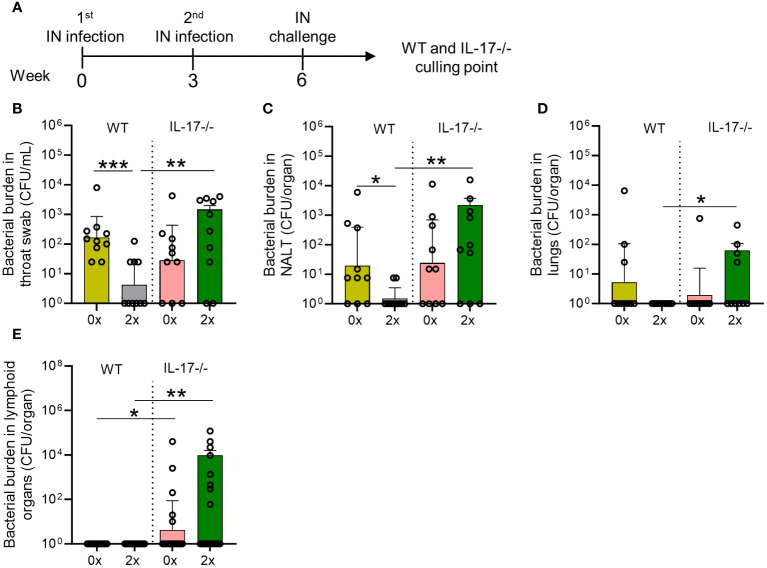
Role of IL-17 in mucosal protection following sequential intranasal infection. **(A)** IL-17-/- and WT BALB/c mice (n = 10, male and female, 4–6 weeks old) were given 2x infections with 2031 (*emm*1), 3 weeks apart. Three weeks later, all mice received a homologous IN challenge. Mice were sacrificed on day 2 post IN challenge to assess the bacterial load in **(B)** throat swab, **(C)** NALT, **(D)** lungs, and **(E)** lymphoid organs. The data are shown as the geometric mean ± geometric SD. Significance was determined using Mann–Whitney rank analysis comparing the sequentially infected IL-17^−/−^ mice with their corresponding infected or naïve WT controls, *p <0.05, **p <0.01, ***p <0.001. The x-axis shows the number of infections before the challenge. 2× received infection at weeks 0 and 3. 0× received only the challenge.

To assess the importance of IL-17 in mucosal immunity against *S. pyogenes* IN infection, we compared IL-17 knockout (IL-17^−/−^) mice with wild-type (WT) BALB/c mice receiving no (0x) or 2x IN infections prior to the final IN challenge (**Figure 3A**). IL-17^−/−^ mice sequentially infected prior to challenge demonstrated significant increases in bacterial burden in throat swabs (p <0.01), NALT (p <0.01), lungs (p <0.05), and lymphoid organs (p <0.01) compared to 2x infected WT mice (**Figures 3B–E**). Sequentially infected IL-17^−/−^ mice showed an increased bacterial burden in the NALT (1.71-fold increase, non-significant), throat swab (3.04-fold increase, non-significant), and lymphoid organs (4.53-fold increase, non-significant) compared to control mice (IL17-/- 0x) (**Figures 3B–E**). In contrast, sequentially infected WT mice showed a reduction in bacterial burden in NALT (p <0.05), lungs (non-significant), and throat swabs (p <0.001) (**Figures 3B–D**). As shown previously (**Figure 1E**), the young and immunocompetent mice did not develop systemic infection post-challenge (**Figure 3E**).

**Figure 4:**
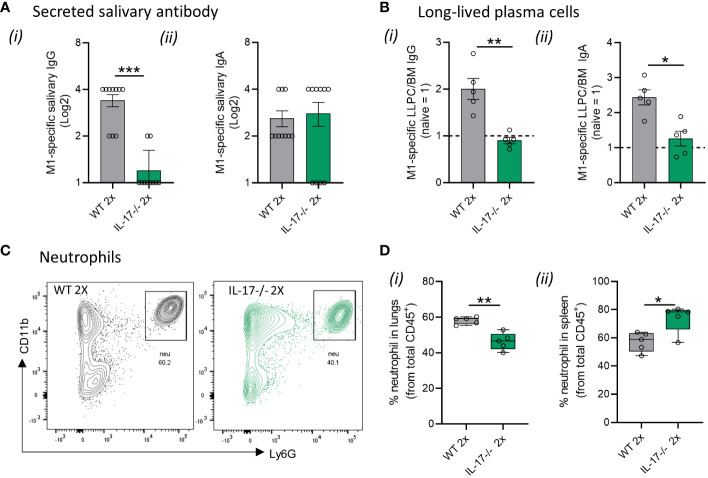
IL-17-mediated immune mechanisms in mucosal protection. IL-17^−/−^ and WT BALB/c mice (n = 5–10, male and female, 4–6 weeks old) were given two IN infections with 2031 (*emm*1), each 3 weeks apart. Three weeks later, all mice received a homologous IN challenge. **(A)** Secreted salivary antibody in IL-17^−/−^ mice. Saliva was collected 7 days following 2x IN to determine M1-specific total **(i)** IgG and **(ii)** IgA titers using ELISA. Data shown are antibody titers in saliva from 2× sequentially infected WT and IL-17^−/−^ mice. **(B)** Quantification of M1 specific **(i)** IgG and **(ii)** IgA LLPC in the bone marrow were enumerated by ELISpot using 3–5 mice/group. Data are presented as number of LLPC per 10^7^ bone marrow (BM) cells obtained from 2× sequentially infected WT and IL-17^−/−^ mice **(C, D)** Neutrophils in lungs and spleen. Representative contour plots from flow cytometry analysis of neutrophils (CD45^+^CD11b^+^Ly6G^+^) in the lungs of WT mice and IL-17-/- mice receiving 2x infections with 2031. **(D)** Data are shown as box-and-whisker plot representing the percentage (%) of neutrophils from CD45^+^ immune cells in the **(i)** lungs and **(ii)** spleen from IL-17 2× sequentially infected WT and IL-17^−/−^ mice. Significance was determined using Mann–Whitney rank analysis. *p <0.05, **p <0.01, ***p <0.001, comparing naïve IL-17^−/−^ and 2× IN infected IL-17^−/−^ mice.

The higher susceptibility to *S. pyogenes* mucosal infection in IL-17-/- mice was due to impaired humoral and cellular responses. This is evidenced by the significantly lower (p <0.001) M1-specific IgG antibodies in the saliva of 2x infected IL-17^−/−^ mice compared to WT mice (**Figure 4Ai**). No difference in M1-specific salivary IgA levels was observed between the sequentially infected IL-17^−/−^ and WT mice (**Figure 4Aii**). The number of M1-specific IgG^+^ and IgA^+^ LLPC in the BM of IL-17^−/−^ mice that received 2x infections, was significantly lower than that in WT mice (p <0.05–0.01) (**Figures 4Bi, ii**, respectively). In contrast to the reduction observed in WT mice, M1-specific IgG titers did not change in the sera of IL-17^−/−^ mice after the 2x infections (**Supplementary Figure 2A**). No changes in IgA responses were observed in the sera of either WT or IL-17^−/−^ mice (**Supplementary Figure 2B**). The role of IL-17 in cell-mediated immunity was assessed in the lungs and spleen of sequentially infected mice. IL-17^−/−^ mice that received 2x infections showed a significant decrease in Ly6G^+^ neutrophils in the lungs (**Figures 4C, Di**), paralleled by an increase in the spleen (**Figure 4Dii**), when compared to the respective WT control. This suggests that mice with intact IL-17 are able to recruit neutrophils from the spleen into the lungs to combat mucosal infection.

Taken together, these data show that the absence of IL-17 prevents the development of humoral and cellular immunity against *S. pyogenes* in the respiratory tract.

### Repeated IN infections can partially prevent systemic dissemination of *S. pyogenes* after skin challenge

**Figure 5:**
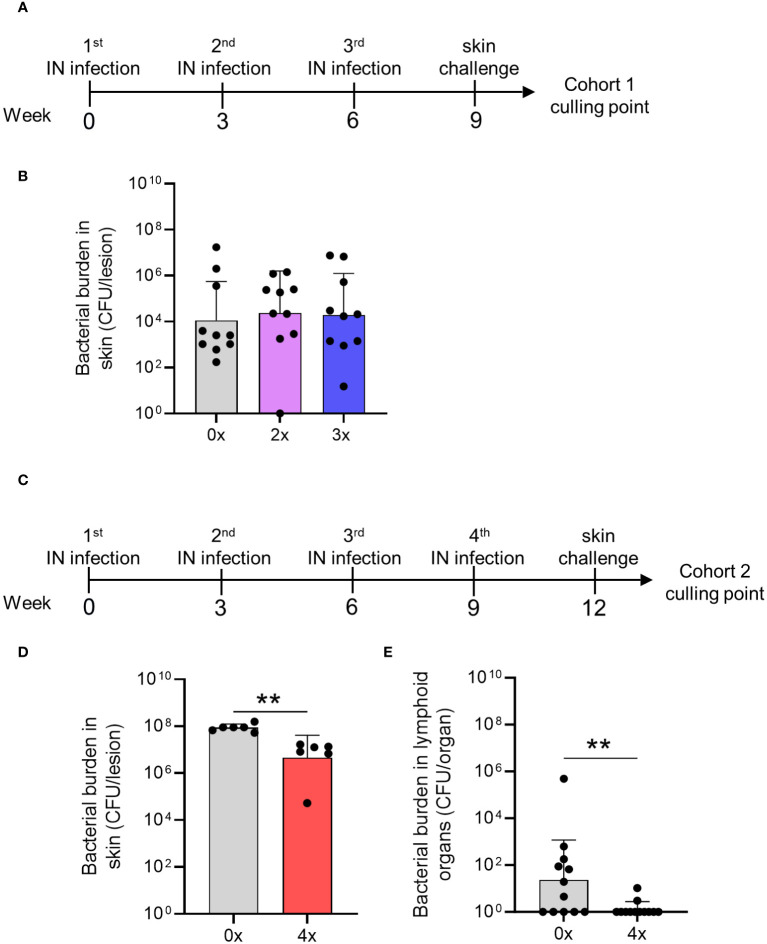
Cross-compartmental protection at the skin following 0–4 prior intranasal homologous infections. **(A)** Cohort 1—mice received homologous superficial skin challenge three weeks following 0x, 2x or 3x infections. BALB/c mice (n = 10, female, 4–6 weeks old) were given IN infections with 2031 (*emm*1), 3 weeks apart. **(B)** Mice were sacrificed on day 6 post skin challenge and skin tissues. Skin CFU for the entire skin lesion are presented **(C)** Cohort 2—mice received 4x infections, 3 weeks apart prior to a homologous skin challenge. **(D, E)** Mice were sacrificed on day 6 post skin challenge to assess bacterial load (CFU) in the skin lesion and lymphoid organs. **(D)** Skin CFU are calculated for the entire skin lesion, and **(E)** CFU in lymphoid organs combined CFU of spleen and pooled cervical lymph nodes for each mouse. The data are shown as the CFU geometric mean ± geometric SD. Significance was determined using Mann–Whitney rank analysis of CFU in sequentially infected group compared to CFU of the control, **p <0.01. The x-axis shows the number of infections prior to challenge. 2× received infection at weeks 0 and 3, 3× at weeks 0, 3, and 6, and 4× at weeks 0, 3, 6, and 9. 0× only received the challenge.

Next, we modeled a scenario in streptococcal endemic settings to assess whether sequential IN infections could provide cross-compartmental protection to the skin and prevent systemic dissemination to lymphoid organs. We have previously demonstrated that at least two sequential homologous skin infections are required to generate skin immunity against a homologous skin challenge (5). In the current study, WT mice were given either 2x or 3x homologous IN infections 3 weeks apart or left naïve (cohort 1). Three weeks after the last IN infection, all mice received a skin challenge with the homologous isolate 2031 (**Figure 5A**). Six days after the skin challenge, the mice in each cohort were euthanized to assess the bacterial burden in various tissues. We found that none of the mice that received 2x or 3x sequential IN infections were protected from the skin challenge (**Figure 5B**). This was evident from the bacterial burden observed in the skin lesions of sequentially infected mice, which was comparable to that observed in control mice (**Figure 5B**). To further investigate whether a higher number of prior IN exposures could induce cross-compartmental protection in the skin, a second cohort of mice (cohort 2) received 4x infections, each 3 weeks apart, following which, 3 weeks later, they received a skin challenge (**Figure 5C**). The mice showed a significant reduction (p <0.01) in the bacterial burden in the skin (95%) and lymphoid organs (95%) compared to control mice (**Figures 5D, E**).

Therefore, cross-compartmental protection against *S. pyogenes* infection is hard to achieve and requires at least four previous homologous exposures.

### Activation of immune responses in the spleen mediate cross-compartmental protection against *S. pyogenes*

**Figure 6:**
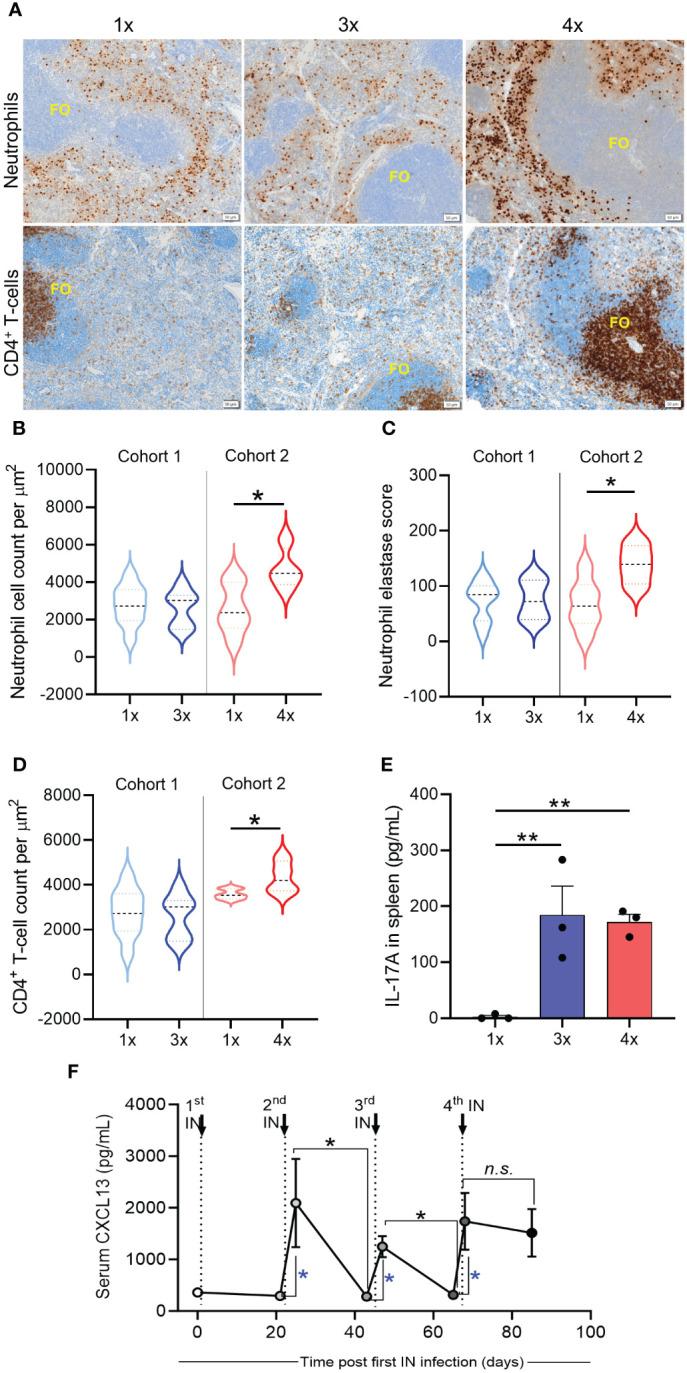
Immune mechanisms associated with cross-compartmental protection. BALB/c mice (n = 5–10, female, 4–6 weeks old) were given repeated IN infections with 2031 (*emm*1), 3 weeks apart. Mice were rested for 3 weeks prior to receiving a homologous skin challenge. **(A)** Representative immunohistochemistry (IHC) images of neutrophils and CD4^+^ T cells. Spleen samples from mice with different number of IN infections (1×, 3x, 4x) were formalin-fixed, paraffin-embedded (FFPE) for IHC. Samples were stained with anti-neutrophil elastase and anti-CD4 for the identification of neutrophils and CD4^+^ T cells, respectively, and counterstained with hematoxylin. Splenic follicles (FO) indicated in the figure. Scales bar = 50 µm. Images representing 1x and 3x were obtained from cohort 1 and images representing 4x from cohort 2. **(B–D)** Enumeration of neutrophils and CD4^+^ T cells. **(B)** Total counts for neutrophils, **(C)** neutrophil elastase H-score, and **(D)** total counts for CD4^+^ T cells were obtained from entire spleen section. Positive cell detection was used to generate a H-score in QuPath. Cell counts were normalized by total tissue area (um^2^). Data are shown as violin plots depicting distribution of numerical data against number of infections. Significance was determined using Mann–Whitney in sequentially infected group compared to 1x infected, *p <0.05. **(E)** IL-17 production by spleen cells was assessed using cytokine bead array (CBA). Splenocytes were stimulated *ex vivo* during 72 h with heat killed 2031 and the supernatant collected for determining the concentrations of IL-17. Data from duplicates are presented as pg/mL mean + SEM. The x-axis shows the number of infections prior to challenge. One Way ANOVA analysis in sequentially infected group compared to 1x infected, **p <0.01 **(F)** Quantitative assessment of GC response. Sera were collected on day 3 and on day 20 post each infection to define concentration of CXCL13. Dotted lines indicate time that 2^nd^, 3^rd^, and 4^th^ IN infections were given. Significance was determined using Mann–Whitney to compare day 3 with day 20 in each infection group, *p <0.05, n.s. = non-significant. Blue * is comparing day 0 (before infection) with day 3 after each sequential infection, *p <0.05. The x-axis shows the number of infections prior to challenge. 1x only received infection at week 3, 3x at weeks 0, 3, and 6, and 4x at weeks 0, 3, 6, and 9.

To understand whether skin and systemic immunity observed after repeated IN infections are associated with the induction of immune responses in the spleen, we analyzed splenic CD4^+^ T cells and neutrophils using immunohistochemistry (IHC) staining. No difference was observed between mice that received 1x or 3x IN infections (**Figures 6A, B**). However, when mice received 4x infections, a significant increase (p <0.05) in the number of neutrophils and neutrophil elastase H-score (a surrogate marker for neutrophil extracellular trap formation (30)) was observed in comparison to mice that received 1x infection (**Figures 6A–C**). While neutrophils accumulated in the perifollicular spaces, increased numbers of CD4^+^ T cells were observed in the splenic follicles of mice that received 4x infections (**Figures 6A, D**). Interestingly, a significant increase in IL-17 secretion by splenocytes stimulated *ex vivo* with HK *S. pyogenes* 2031 was observed after 3x IN infections (**Figure 6E**); however, it did not correlate with protection from skin challenge (**Figures 5B**). CD4^+^ T cell accumulation in splenic follicles led to the hypothesis that germinal center responses may play a role in cross-compartmental protection. To confirm this, we investigated plasma CXCL13 levels. CXCL13 is a B cell chemoattractant used as a plasma marker of germinal center activity (31) and has also been shown to play a role in mucosal immunity (32). We assessed the level of CXCL13 in mouse sera collected three days and three weeks after each homologous sequential IN infection (**Figure 6F**). A significant transient increase in serum CXCL13 levels was noted at 3 days post 2^nd^, 3^rd^, and 4^th^ IN infections (4.4- and 5.5-fold increase, respectively); however, at 3 weeks post 2^nd^ and 3^rd^ infection, CXCL13 levels had dropped to levels comparable to those in the naive sera. However, following the 4^th^ IN infection, significantly increased CXCL13 levels were maintained for at least until 3-weeks post infection (**Figure 6F**). Notably, in these mice, a significant increase in CXCL13 levels following 2nd infection also coincided with an increase in serum IgG (**Supplementary Figure 1**). Although CXCL13 demonstrated a transient, albeit significant, increase following each IN infection, serum IgG titers remained consistent.

Taken together, repeated URT mucosal infections generated cross-compartmental immunity in the skin and spleen, which was not associated with changes in IL-17 levels but rather correlated with neutrophil infiltration and germinal center activity, as evidenced by increased levels of serum CXCL13 and expansion of CD4^+^ T cells in the splenic follicles.

## Discussion

The mechanisms governing natural immunity to *S. pyogenes*, a major human pathogen associated with high morbidity and mortality, are poorly understood due to the diversity of the pathogen and the various infection sites it targets. In this study, we modeled streptococcal exposure in a natural scenario to elucidate the immune mechanisms underlying protection of the respiratory mucosa and cutaneous (skin) sites. Our findings demonstrate a dichotomous role of IL-17 in protection against streptococcal infections. IL-17 plays a crucial role in preventing the systemic dissemination of *S. pyogenes*, which colonizes the respiratory mucosa. To our knowledge, this is the first study to demonstrate that a single IN infection with *S. pyogenes* can lead to protective immunity against a homologous reinfection in the respiratory mucosa, associated with an early induction of IL-17 secretion by lung cells. Conversely, systemic immunity following 4x IN infections was independent of IL-17 production by spleen cells and relied on germinal center formation and neutrophil recruitment.

IL-17 is a pro-inflammatory cytokine that exerts protective effects against bacterial and fungal infections. It is rapidly released in response to specific triggers on mucosal surfaces. IL-17 contributes to maintaining epithelial homeostasis, recruiting neutrophils, and stimulating T cell-dependent B cell responses, and acts as a crucial link between innate and acquired immune responses (33). Mice deficient in the IL-17 receptor have increased susceptibility to mucoepithelial bacterial infections (34, 35). Additionally, the kinetics of IL-17 generation in the lungs appears to be important. In the context of safeguarding against lung infection with *Klebsiella pneumoniae*, the initial 12 h–24 h post-infection has been demonstrated to be critical for neutrophil recruitment, as well as for the optimal expression of granulocyte colony-stimulating factor and macrophage-inflammatory protein-2 (36). Furthermore, reduced IL-17 production has been linked to increased bacterial dissemination and diminished survival of *Citrobacter rodentium* (37), *Mycoplasma pneumoniae* (38), and *Porphyromonas gingivitis* (39). In all these infection models, increased susceptibility resulting from IL-17 deficiency is associated with reduced early neutrophil infiltration into the infected tissue. Moreover, a study by 17 suggested that IL-17 may augment neutrophil bactericidal activity. Taken together, these findings highlight that the progression of IL-17 is of critical importance in vaccine design. These results align with our data, indicating that following a single IN infection with *S. pyogenes*, IL-17 plays a critical role in protecting mice from subsequent infections and prevents dissemination into systemic sites. Furthermore, we found that IL-17 deficiency led to *S. pyogenes* escaping from the respiratory tract into sterile sites, demonstrating its importance in preventing invasive streptococcal disease originating at mucosal sites. This was likely due to the inadequate production of M protein-specific IgG antibodies in saliva and impaired recruitment of effector immune cells to the respiratory mucosa.

Furthermore, we identified the prerequisites for mucosal and systemic protection following repeated IN infections. Previous studies in mice have reported the ability of repeated infections with *S. pyogenes* to develop mucosal immunity (8, 40). However, the number of infections required to induce immunity and the duration of protective immune responses remain unclear. We found that a single mucosal infection occurring 3 weeks prior to a homologous challenge generated *S. pyogenes* type-specific immunity. Given over 250 *emm* types, achieving pan-streptococcal immunity will likely take several years or may never fully occur following a natural infection. This explains the high incidence of streptococcal skin and mucosal infections in children under the age of 10, which decreases in early adulthood (41). Furthermore, we have previously demonstrated age-related acquisition of antibodies to the conserved region of *S. pyogenes* M-protein in teenagers and adults residing in endemic areas (42) and demonstrated that vaccination of mice with conserved region peptides can induce protection from both mucosal and skin challenges (43–45).

Understanding the mechanisms and longevity of immune responses following infection is of paramount importance for developing effective public health measures against infectious diseases. Our study investigated immunity at various anatomical sites of *S. pyogenes* infection. Remarkably, we observed that mice developed mucosal immunity after a single IN infection, which persisted for at least 9 weeks. In contrast, our previous findings following S*. pyogenes* skin infection showed that mice required reinfection with the same strain to establish long-lasting immunity (5). These findings emphasize the existence of distinct immune mechanisms associated with different infection routes. Epidemiological evidence from Australian communities with recurrent pyoderma suggests that repeated skin exposure confers immunity against throat infections (46–48). Nonetheless, it is important to note that repeated skin infections have also been implicated in the development of rheumatic fever (49). Our study aimed to investigate the lack of protection against a single skin infection and assess whether multiple mucosal exposures could provide immunity to the skin and prevent systemic dissemination of *S. pyogenes*. We found that mice required a minimum of 4x homologous mucosal infections to achieve significant protection in the skin and lymphoid organs, underscoring the challenges associated with developing multi-site immunity (50–52).

Only after the fourth IN infection did the levels of CXCL13 become sustained in the plasma. CXCL13 is expressed in lymphoid follicles and acts as a chemotactic signal for CXCR5 expressing B- and T cells (32). CXCL13 has been implicated in mucosal immunity by promoting germinal center formation, facilitating Ig isotype switching (32), and activating macrophages *via* IL-17-dependent mechanisms (5, 53, 54). Accordingly, we observed a modest increase in M-type specific serum IgG after 2x infections, coinciding with the initial increase in CXCL13 systemic levels. Evidence suggests that antibodies produced in response to primary skin infection can confer type-specific immunity against future streptococcal skin infections (5, 14, 55). Interestingly, protective immunity in the respiratory mucosa did not rely heavily on germinal center activation or circulating antibodies, as mice with low levels of M-specific IgG antibodies were still protected after a single infection. Low levels of circulating M-type-specific antibodies during active infection are unsurprising, potentially stemming from IgG cleavage by specific proteases or binding to *S. pyogenes*, followed by subsequent removal (56). Interestingly, we observed an increase in IgG- and IgA-secreting cells in the bone marrow following sequential IN infections, consistent with our previous findings that *S. pyogenes* skin infections gradually increased the number of M-type-specific antibody-secreting cells in the bone marrow, which led to increased protection (5).

After initial priming (first mucosal infection), we observed a significant influx of neutrophils in the lungs, which likely led to bacterial killing via antibody-mediated phagocytosis (57). This was paralleled by an increase in effector/memory CD4^+^ T cells in the lungs. This population contains both long-lived lung resident memory T cells that mediate immunity against *S. pyogenes* mucosal infection (44, 58) and short-lived effector T cells, which recirculate between the blood, lymphatics, or other peripheral tissues and die after acute infections (59, 60). The markers used to identify EM CD4^+^ T cells in this study were not sufficient to distinguish short-lived effector T cells without memory potential from long-lived memory cells. A subset of lung CD4^+^ effector T cell producers of IL-17 (Th17 cells), has been shown to effectively support B cell responses and induce a pronounced IgG antibody response (61), corroborating our findings of disrupted M-specific IgG antibodies in the saliva and BM of IL-17^−/−^ mice. While most IgG in saliva is derived from the blood circulation by passive leakage, dimeric IgA is produced in the stroma of the salivary glands. IL-17A is vital for the generation of salivary IgA (36) and protects against *S. pyogenes* (18) and other bacterial mucosal infections (62, 63). In the current study, WT mice generated M1-specific antibodies proportional to the number of mucosal exposures, while IL-17^−/−^ mice had significantly lower IgG antibodies, which correlated with a lack of protection. The protective role of IL-17 through recruitment of neutrophils and tissue-resident memory T cells is evident in our study, as IL-17^−/−^ mice had reduced numbers of lung neutrophils and effector/memory CD4^+^ T cells, as also shown elsewhere (63–65). Instead of generating immunity, repeated IN infections in IL-17^−/−^ mice led to *S. pyogenes* accumulation in the respiratory mucosa and the invasion of sterile sites. This could be attributed to the fact that in the absence of IL-17, *S. pyogenes* could not be cleared from the respiratory mucosa and accumulated post-infection. IL-17A is vital for maintaining the integrity of the epithelial barrier (66).

In summary, our findings highlight that IL-17 orchestrates a multifaceted mechanism required to induce immunological memory and neutrophil recruitment to the lungs, preventing the subsequent systemic dissemination of *S. pyogenes* following mucosal infections. However, it does not prevent *S. pyogenes* infections that begin at the skin site. Our study demonstrates that cross-compartmental protection is challenging to achieve with natural infections. This highlights the importance of developing vaccine strategies that lead to timely induction of cellular and humoral responses capable of protecting multiple anatomical sites from *S. pyogenes* infection. Understanding the molecular and cellular basis of mucosal and systemic immune responses will provide important insights into the rational design of effective vaccines to prevent superficial and invasive streptococcal diseases.

## Materials and methods

### Ethical statement

Mice were housed at the Animal Facility of Griffith University (Gold Coast, Australia). All experiments and animal procedures were approved by the Griffith University Animal Ethics Committee (Animal Ethics Approval GLY/04/18), in compliance with the Australian National Health and Medical Research Council Guidelines. Experimental protocols involving IL-17 knockout (IL-17^−/−^) mice were reviewed and approved by the Office of the Gene Technology Regulator (OGTR). BALB/c mice (female, 4–6 weeks old) were sourced from the Animal Resource Centre, Western Australia. IL-17^−/−^ mice were obtained from Yoichiro Iwakura (Tokyo University of Science, Japan) under a Material Transfer Agreement (MTA) (67). Knockout mice were bred in-house at the Griffith University Animal Facility. The general health of the mice was monitored daily. Following the challenge, mice were monitored for signs of illness as per a score sheet approved by the Griffith University Animal Ethics Committee (68). The observer was blinded to the experimental groups.

### Bacterial strains and culture media

*S. pyogenes* 2031 (*emm*1) was obtained from the Menzies School of Health Research (Darwin, NT, AUS). The isolate was serially passaged in mice to ensure virulence and was resistant to 200 µg/ml of streptomycin. The isolate was grown overnight in liquid Todd–Hewitt broth medium (THB; Oxoid, AUS), supplemented with 1% yeast and 1% neopeptone (THBYN; Difco, AUS). The isolate was 10-fold serially diluted and plated in duplicate on Columbia Blood Agar (CBA; Oxoid, UK) supplemented with 5% defibrinated horse blood (Equicell, AUS) and 200 µg/ml streptomycin (Sigma, China) to determine the number of colony-forming units (CFUs).

### Pepsin extraction of the M protein

M-protein was extracted using pepsin digestion as described previously (69, 70). Overnight THBYN cultures were pelleted and resuspended in four times the weighted volume of PBS pH 5.8 twice. Resuspended pellets were pre-warmed to 37°C for enzymatic digestion with pepsin A (Merck, AUS) (1 mg pepsin per 10 g bacterial suspension) for 45 min with intermittent mixing. The digested suspension was pelleted, and the pepM extract buffer was exchanged with PBS pH 7 using a 10 kDa Amicon centrifugal filter unit (Merck, Ireland). PepM extracts were confirmed by SDS-PAGE and stored in solution at −20°C.

### Sequential intranasal infection protocol

The mice received sequential intranasal infections 3 weeks apart. Mice were anesthetized via an IP injection of ketamine 100 mg/kg and xylazine 20 mg/kg (71). Using a pipette, 5 µL of bacterial inoculum was administered to each nare (1 × 10^7^ CFU in 10 µL/mouse) while the mouse remained on its back to ensure inhalation. Mice were monitored daily as described above.

### Superficial skin challenge

Mice were challenged using a skin scarification model as previously described (72). The mice were anesthetized with an IP injection of ketamine 100 mg/kg and xylazine 20 mg/kg. The fur from the neck nape was removed using clippers and then superficially scarified. A 10 µL inoculum was topically applied; once the inoculum had absorbed into the skin, a temporary cover (Band-Aid™) was applied to the wound, and mice were housed individually. The mice were monitored daily for signs of illness, as described above.

### Antibody response by indirect enzyme-linked Immunosorbent assay (ELISA)

Indirect-ELISA was used to quantify antigen-specific IgG and IgA antibody titers as described elsewhere (73). Goat anti-mouse IgG (Bio-Rad, AUS) or IgA (Invitrogen) horseradish peroxidase (HRP) linked antibodies were used to detect antigen-specific antibodies. Optical density (OD) at 450 nm was measured using a Tecan Infinite m200 Pro plate reader. Titers were defined as the highest dilution of serum for which the OD was >3 standard deviations (SD) above the mean OD of the control samples (naïve sera).

### Sample collection and CFU quantification

Serum samples were collected by puncturing the submandibular vein. Whole blood was allowed to clot and be removed prior to centrifugation for serum separation.

Throat swabs were performed using floq swabs (Interpath, USA) moistened in PBS prior to swabbing both sides of the throat. Swabs were squeezed into tubes containing PBS, 10-fold serially diluted, and plated onto CBA plates supplemented with 5% horse blood and 200 µg/ml of streptomycin. Following overnight incubation at 37°C, the CFU were counted to determine the bacterial load.

At designated time points, the mice were sacrificed via CO_2_ inhalation, and whole blood was collected via cardiac puncture into tubes containing ethylenediaminetetraacetic acid (EDTA; Thermo, AUS). Tissues were collected and mechanically homogenized using a Bullet Blender™ (Next Advance, USA) following the manufacturer’s instructions. Samples were 10-fold serially diluted and plated in replicates on CBA plates supplemented with 5% horse blood and 200 μg of streptomycin. Following overnight incubation at 37°C, CFU were counted to determine bacterial load.

### Antibody-secreting cell response by enzyme-linked immunosorbent spot assay (ELISpot)

ELISpot was used to quantify the number and location of antibody-secreting cells (ASC) in the splenocytes, lungs, and long-lived plasma cells (LLPCs) in the bone marrow. Multiscreen HA filter plates (Merck, Ireland) were coated with 5 μg/mL M1 extract in carbonate coating buffer overnight at 4°C. Isolated cells were adjusted to 5 × 10^6^/mL and directly tested for IgG/IgA-secreting cells using previously published methods (74, 75). Spots were developed using an AEC substrate kit (BD, AUS) according to the manufacturer’s instructions and manually counted to determine ASCs.

### CXCL13 assay

The CXCL13 assay was performed according to the manufacturer’s instructions (R&D Systems, USA). Test sera were diluted 1:4 and assay standards were prepared in a 1:1 ratio of assay diluent in duplicate and incubated at room temperature for 2 h on a shaker. The plate was washed five times before conjugate incubation at room temperature for 2 h on a shaker. After washing, the substrate solution was added for 30 min at room temperature in the dark prior to the addition of the stop solution. The OD was measured at 450 nm with a correction of 540 nm using a Tecan Infinite m200 Pro plate reader.

### IL-17 ELISA

IL-17A ELISA was performed according to the manufacturer’s instructions (Mouse IL-17A ELISA MAX; Biolegend, USA). Nunc MaxiSorp plates (Thermo, AUS) were coated overnight with an IL-17A capture antibody diluted in carbonate coating buffer. The plates were blocked with 1% BSA/PBS for 1 h prior to washing. Splenocyte supernatants (diluted 1:4 in duplicate) and standards were incubated at room temperature for 2 h. The detection antibody was incubated for 1 h prior to avidin–HRP incubation for 30 min. TMB substrate solution was incubated for 20 min, followed by the addition of an acid stop solution. The absorbance (OD) was measured at 450 nm with a correction of 570 nm. IL-17A concentration was determined by plotting the unknown samples on a standard curve to determine IL-17A pg/mL.

### Cell purification for *ex vivo* assays

Lymphocyte populations were purified from the splenocytes, lungs, and bone marrow. Lungs were digested in Worthington collagenase III (Scimar, AUS) supplemented with DNase I (Merck, AUS), and all tissues were transferred through a 0.70 μM cell strainer (Corning, USA) to obtain single-cell suspensions. RBCs were lysed with ACK lysis buffer and washed with RPMI (Thermo Fisher Scientific, AUS).

### Cytokine production by cytometric bead CBA analysis

Single cell suspensions of splenocytes were prepared as described above, counted using a hemocytometer in trypan blue (Sigma, AUS) and adjusted to 4 × 10^6^ cells/mL. Splenocytes were stimulated with heat-killed 2031 or medium as a negative control. After 72-hour stimulation at 37°C with 5% CO_2_, the cells were centrifuged at 300*g* for 10 min, and the supernatant was collected. The supernatants were stored at −80°C. Cytokines in the supernatant were subsequently measured using a mouse Th1/Th2/Th17 CBA kit (BD, AUS) according to the manufacturer’s instructions. Samples were acquired using a BD LSR Fortessa cytometer, and data were analyzed using FCAP Array v3.1 (BD).

### Cell population analysis by flow cytometry

The cell populations were determined by flow cytometry. Single cell suspensions were prepared as described above and pre-incubated with the Fc block (CD16/32) for 15 min on ice. The cells were surface-stained with a master mix containing dead cell exclusion dye (NIR), CD4-FITC, CD62L-PE-Cy7, CD44-APC, CD45-BUV395, CD11b-BUV737, CD3-PE-CF594, and Ly6G-BV510. Cells were stained on ice in the dark for 40 min. Following incubation, the cells were washed in FACS buffer (2.5% fetal calf serum, 5mM EDTA in PBS) and fixed in 2% paraformaldehyde for 15 min. Samples were washed in PBS and acquired on a BD LSR Fortessa flow cytometer, and data were analyzed using FlowJo V.10.7 (BD).

### Immunohistochemistry (IHC)

Formalin-fixed paraffin-embedded (FFPE) spleens were sectioned at 3 μm thickness on SuperFrost+ slides. IHC staining of spleen sections for CD4 (1:200, D7D2Z; Cell Signaling Technology) and neutrophil elastase (1:200, E8U3X; Cell Signaling Technology) was performed using a Leica BOND™ RX auto-stainer (Leica, Nussloch, Germany) with the BOND Polymer Refine Detection (Leica) kit and developed with 3,3’-diaminobenzidine as the chromogen. The stained slides were mounted in Dako Mounting Medium (Dako) and coverslipped using a Dako coverslipper.

### IHC acquisition and analysis

Images were acquired using an Olympus VS200 digital slide scanner (EVIDENT Life Science, USA) under bright-field emission. Each tissue section was acquired using a ×20 objective (UPLXAPO ×20; NA 0.8). Individual images were obtained from regions of interest (ROI) drawn using the VS200 software based on automatic outline thresholding. Cell counts were performed using QuPath (v 4.0.1), and the neutrophil elastase H-score was assigned in the range of 0–300. Cell counts are given as the average of cells per µm2Data was exported to Microsoft Excel for tabulation and plotted using GraphPad Prism.

### Statistical analysis

The data were analyzed using Graph Pad PRISM version 10.7 for Windows. All data, except where noted, are presented as the geometric mean ± standard error of the mean (SEM). Statistical differences between the two groups were determined using the non-parametric Mann–Whitney *t*-test. When comparing more than two groups, analyses were performed using one-way ANOVA with *p <*0.05 considered to be statistically significant.

## Data availability statement

The original contributions presented in the study are included in the article/**Supplementary Material**. Further inquiries can be directed to the corresponding authors.

## Ethics statement

The animal study was approved by Griffith University Animal Ethics Committee (Animal Ethics Approval GLY/04/18). The study was conducted in accordance with the local legislation and institutional requirements.

## Author contributions

JLM: Formal analysis, Investigation, Methodology, Writing – review & editing, Writing – original draft. AL: Conceptualization, Formal analysis, Investigation, Methodology, Visualization, Writing – original draft. VO: Investigation, Methodology, Writing – review & editing. JD: Investigation, Methodology, Writing – review & editing. JK: Methodology, Visualization, Writing – review & editing. AC: Investigation, Writing – review & editing. YH: Investigation, Methodology, Writing – review & editing. AH: Methodology, Writing – review & editing. AZ: Conceptualization, Supervision, Writing – review & editing. MG: Conceptualization, Resources, Supervision, Writing – review & editing. MP: Conceptualization, Funding acquisition, Project administration, Supervision, Writing – review & editing, Writing – original draft.
