# Supplementary figures and images for: Disruption of IL-17-mediated immunosurveillance in the respiratory mucosa results in invasive Streptococcus pyogenes infection
Source: Front Immunol. 2024 Mar 21;15:1351777. doi: 10.3389/fimmu.2024.1351777 (PMC10991685; doi:10.3389/fimmu.2024.1351777)

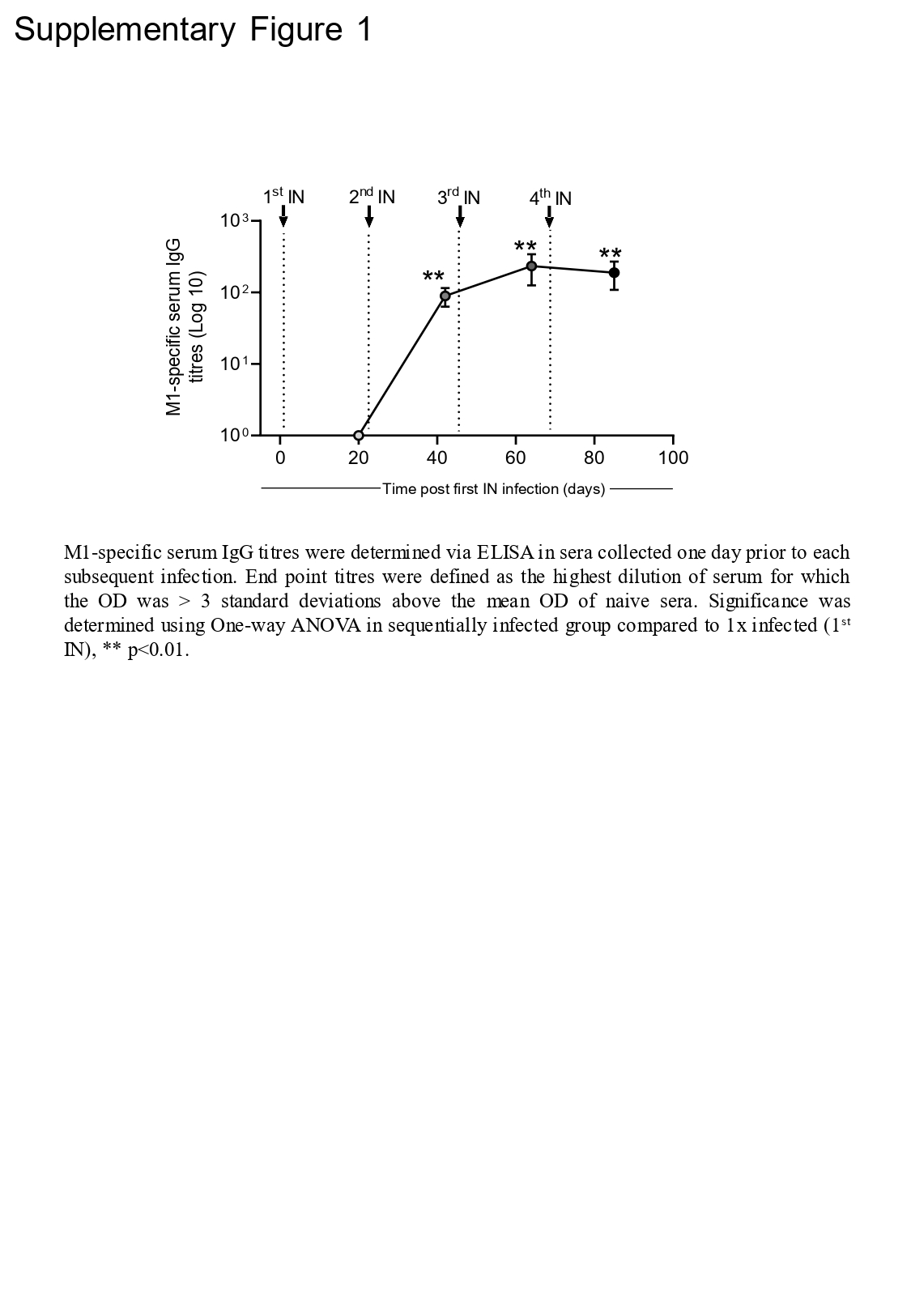

Supplement: Supplementary file 1 [file Image_1.jpeg]

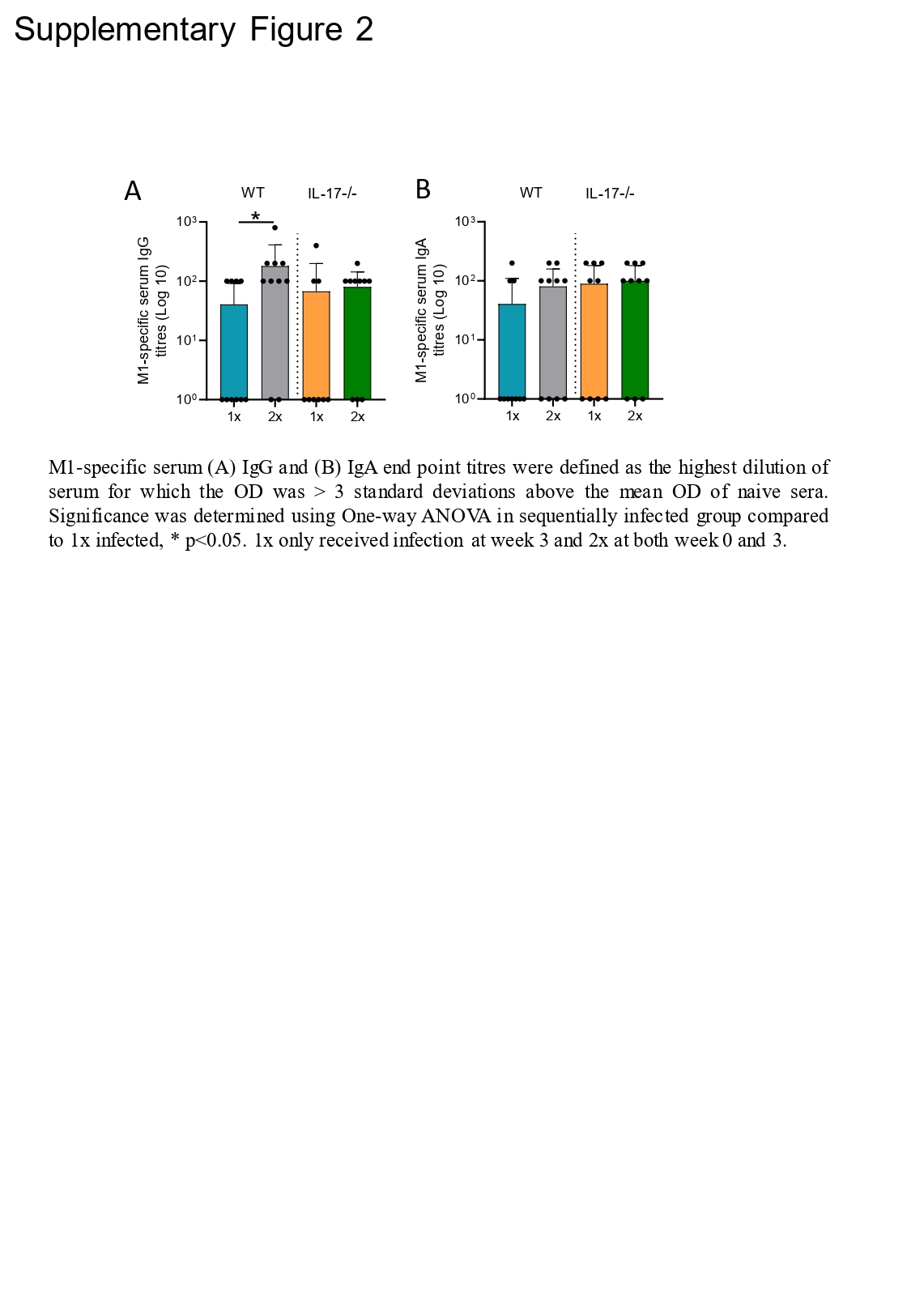

Supplement: Supplementary file 2 [file Image_2.jpeg]
